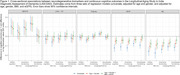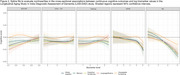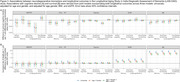# Associations between neurodegenerative plasma biomarkers and cognitive functioning and decline in India

**DOI:** 10.1002/alz70856_099410

**Published:** 2025-12-24

**Authors:** Emma Nichols, Jinkook Lee, Alden L. Gross, Masroor Anwar, Abhishek Gupta, Eileen M. Crimmins, Bharat Thyagarajan, Sharmistha Dey

**Affiliations:** ^1^ University of Southern California, Los Angeles, CA, USA; ^2^ Center for Economic and Social Research, University of Southern California, Los Angeles, CA, USA; ^3^ Johns Hopkins Bloomberg School of Public Health, Baltimore, MD, USA; ^4^ All India Institute of Medical Sciences, New Delhi, Delhi, India; ^5^ University of Minnesota, Minneapolis, MN, USA

## Abstract

**Background:**

Mounting evidence supports the use of blood‐based neurodegenerative biomarkers as a low‐cost, minimally invasive tool for studying dementia, but existing data largely come from clinical samples or high‐income settings. Despite emphasis in the literature on the importance of understanding the utility neurodegenerative biomarkers in diverse populations, published analyses on this topic are limited.

**Method:**

In this observational cohort study, we used nationally‐representative data from the Longitudinal Aging Study in India–Diagnostic Assessment of Dementia (*N* = 2,699) to quantify associations between five neurodegenerative blood biomarkers (β‐amyloid 42/40 [Aβ42/40], total tau, phosphorylated Tau181 [pTau‐181], glial fibrillary acidic protein [GFAP], neurofilament light [NfL]) and both cross‐sectional and longitudinal cognitive outcomes. We used cross‐sectional linear regression models and longitudinal joint models for cognitive decline and mortality, adjusting for age, sex/gender, BMI, and kidney function. We explored non‐linearities and effect modification.

**Result:**

We observed associations between biomarkers and cross‐sectional cognitive functioning (Aβ42/40, GFAP, and NfL) and longitudinal cognitive change (pTau‐181 and NfL). For example, each standard deviation higher observed level of GFAP was associated with a 0.04 (95% Confidence Interval [CI] 0.01 to 0.08) standard deviation (SD) lower general cognitive functioning, similar to the effect of one year of age. Relationships were largely linear in nature, although we observed a threshold effect for GFAP, wherein the association between GFAP and cognition was only present at the highest biomarker levels. We saw little evidence of effect modification by demographic variables or APOE‐ε4 status. NfL had some of the strongest associations across all models; each SD increase in log NfL was associated with 0.006 (95% CI 0.000‐0.013) SD unit/year worse cognitive decline, equivalent to about 35% of the mean longitudinal decline in the sample.

**Conclusion:**

Findings are consistent with prior studies and support the use of neurodegenerative biomarkers in India. Future research should leverage these biomarkers in the Indian context to address a range of research topics, including heterogeneity in dementia phenotypes, links between dementia risk factors and neurodegenerative biomarkers, and interactions between neurodegenerative biomarkers and vascular health in their effects on cognitive outcomes. Biological heterogeneity of dementia and interactions with vascular health warrant further research.